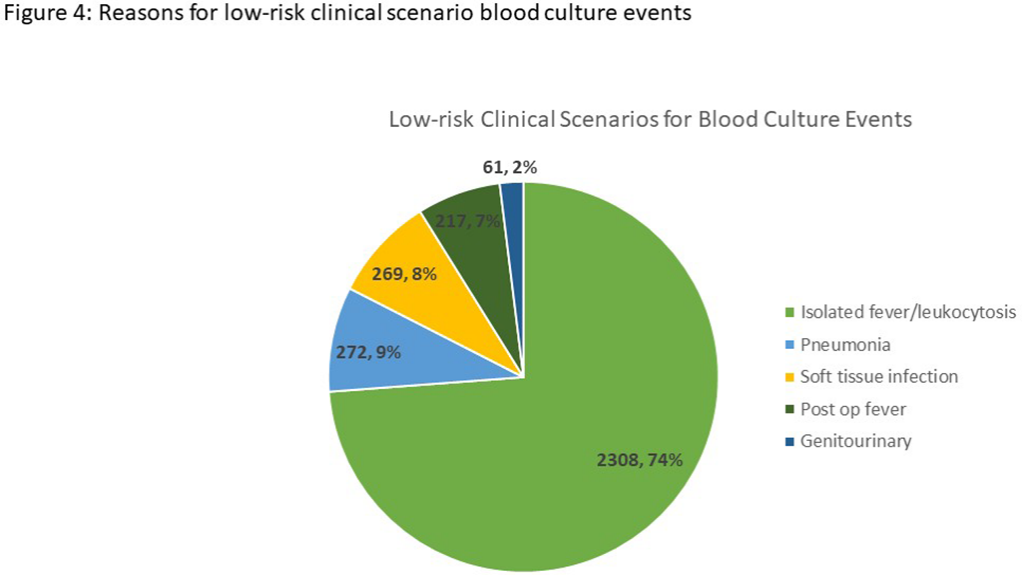# 178 Emerging vancomycin and metronidazole resistance in Clostridioides difficile: Pilot Whole-Genome Sequencing analysis from a multicenter

**DOI:** 10.1017/ash.2026.10576

**Published:** 2026-06-23

**Authors:** Jessica Seidelman, Michael Yarrington, Stefani Dubea, Jonathan Bae

**Affiliations:** 1 Duke University; 2 Duke University Medical Center; 3 Duke University Health System

## Abstract

**Introduction:** In the setting of the BACTEC BCx bottle shortage in 8/2024, we established a clinical standard for BCx stewardship using evidence-based best practices. Specifically, we implemented clinical decision support (CDS) within the electronic medical record (EMR) across the health system to prompt reflection on the indication for BCx collection and discourage low-yield testing. Our primary objective was to compare blood culture event (BCE) rates before (8/2022-7/2024) and after (8/2024-8/2025) CDS implementation. Secondary objectives included comparing all-cause mortality, 30-day readmission, BCx contamination rates, antimicrobial use, central line–associated bloodstream infection rates, and hospital-onset Clostridioides difficile infection rates before and after CDS implementation. **Methods:** This study was conducted within an academic health system comprising 3 inpatient hospitals. In 8/2024, we integrated CDS into the EMR (Epic Systems, Verona, WI). (Figure 1) CDS was displayed whenever a provider ordered a BCx set. Providers were prompted to document the clinical indication for the BCx, stratified as high-, medium-, or low-yield based on the algorithm developed by Fabre et al. in the DISTRIBUTE study. If the selected indication was categorized as low-yield, the CDS displayed a message noting the likelihood of low-diagnostic yield and required additional justification documentation. Providers retained discretion to proceed with the order. We used an interrupted time series (ITS) analysis to compare BCE rates in the pre- versus post-intervention periods. **Results:** The preintervention period had a rate of 52.8 BCE per 1000 inpatient days compared to 46.5 BCE per 1000 inpatient days in the postintervention period (0.88 95% CI 0.87-0.89 p< 0.01). (Figure 2) ITS analysis demonstrated a significant decrease in BCE rate by 17.5% (95% CI -20.7%, 014.2%, P-value < 0.01) at the time of the intervention, but a significant increase in BCE rate of 1.0%% (95% CI 0.6%, 1.4%, p-value <0.01) in the post-intervention period. (Figure 3) We also saw a significant increase in days of antibiotic therapy and BCx contamination rates. (Figure 2) 37,418 BCEs were placed when the CDS was active. Of these, 22,994 BCE (61.5%) were ordered for sepsis. Of the remaining 14,424 BCE, 3,127 (22.0%) occurred for low-risk clinical scenarios. (Figure 4) **Conclusions:** We implemented hospital systemwide CDS during a critical BCx bottle shortage. While we saw an initial decrease in BCE rates, BCE rates increased following implementation. Specifically, many BCE occurred for low-risk clinical scenarios. Thus, while electronic CDS can be helpful, additional feedback mechanisms are necessary for persistent change in BCx stewardship practices.

**Figure:**
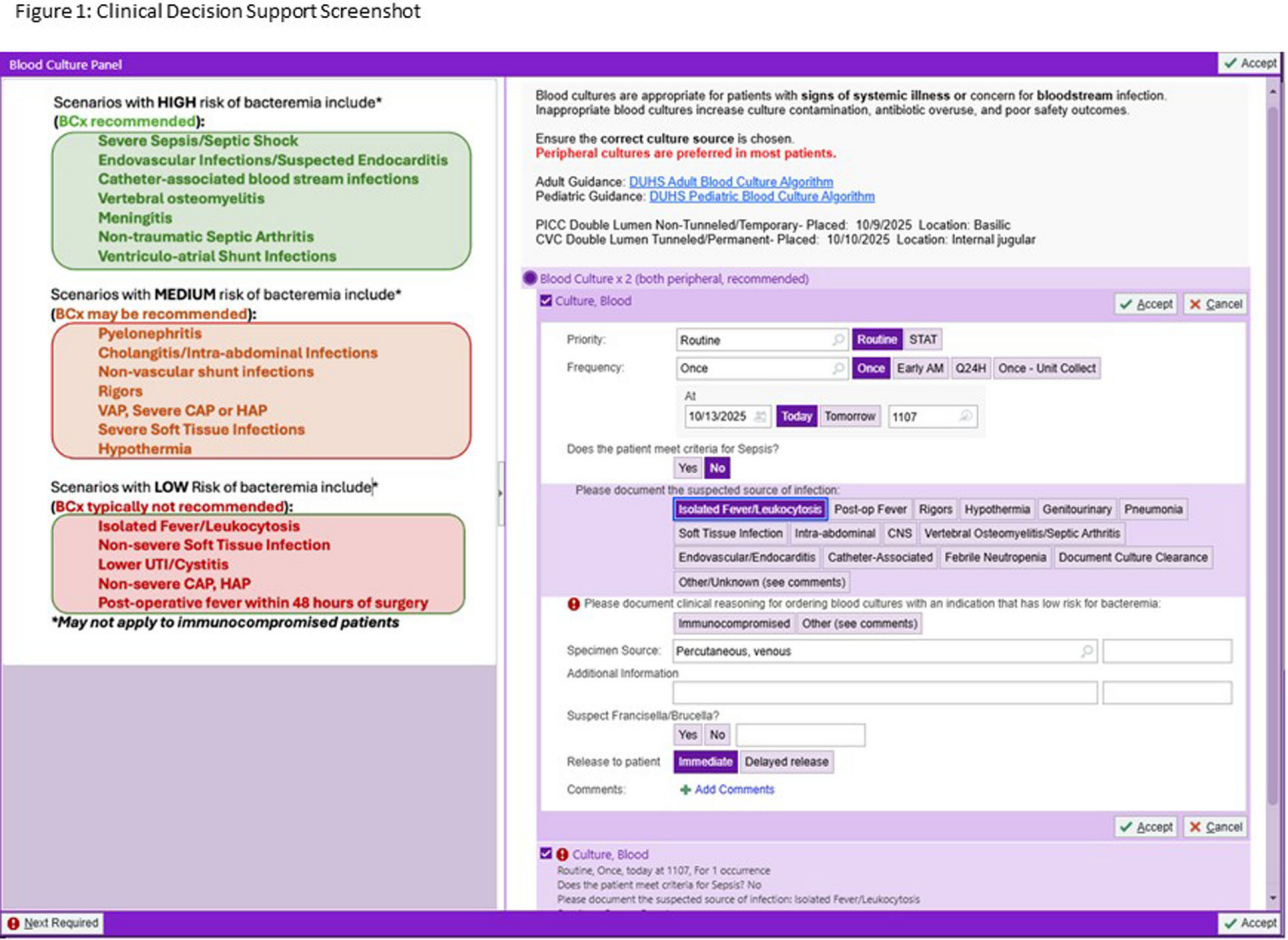

**Figure:**
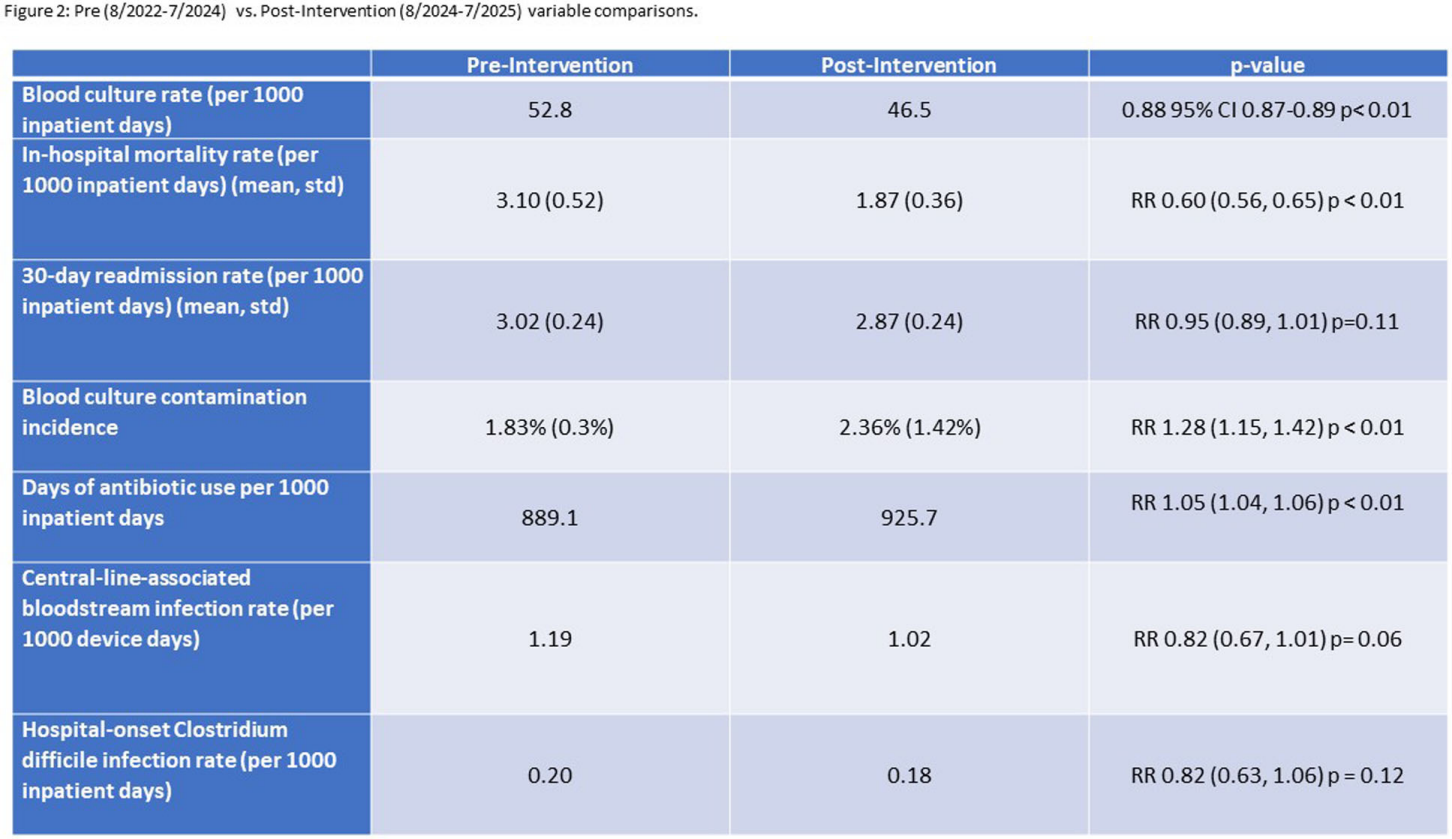

**Figure:**
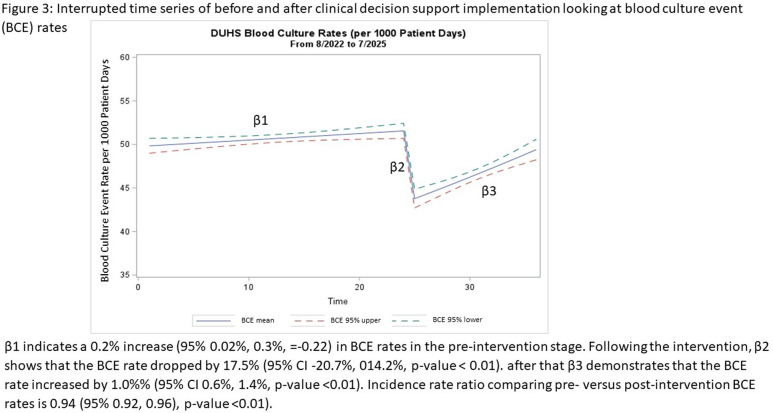

**Figure:**